# Supplementary material for: DEER-PREdict: Software for efficient calculation of spin-labeling EPR and NMR data from conformational ensembles
Source: PLoS Comput Biol. 2021 Jan 22;17(1):e1008551. doi: 10.1371/journal.pcbi.1008551 (PMC7857587; doi:10.1371/journal.pcbi.1008551)
Supplement: S1 Text — (PDF) [file pcbi.1008551.s001.pdf]

Supporting Information for "DEER-PREdict: Software for Efficient Calculation of Spin-Labeling EPR and NMR Data from Conformational Ensembles"

Giulio Tesei[1,◕], João M. Martins[1,◕], Micha B. A. Kunze[1], Yong Wang[1], Ramon Crehuet[1,2], Kresten Lindorff-Larsen[1,*]

1 Structural Biology and NMR Laboratory & the Linderstrøm-Lang Centre for Protein Science, Department of Biology, University of Copenhagen, Copenhagen, Denmark
2 CSIC-Institute for Advanced Chemistry of Catalonia (IQAC), Barcelona, Spain

◕These authors contributed equally to this work.
* lindorff@bio.ku.dk

Molecular Dynamics Simulations

HIV-1 protease

All simulations were performed using GROMACS 5.1 [1] with the PLUMED 2 [2] plugin. Unbiased and RDC-biased metadynamics [3, 4] simulations where performed starting from a closed conformer after removal of the inhibitor from the X-ray crystal structure (PDB code 3BVB) [5]. The simulated protein is the wild type subtype B from isolate BRU/LAI, which differs from the construct of the reference experiments [6] by the following mutations: M36norleucine, S37N, R41pseudo-homoglutamine, M46norleucine, I63P, I64V, A67α-aminobutyric acid and A95α-aminobutyric acid. The protein was simulated in a cubic box with a side length of 8.677 nm containing 22,228 water molecules, 59 sodium cations, and 67 chloride anions. Although the K55C mutations have been shown to have a negligible impact on enzymatic activity [7], we maintained the lysine residues at the spin-labeled positions with the aim of capturing the conformational ensemble of the wild type.

The system was equilibrated for 10 ns with a 2-fs time step in the NPT ensemble, using the Berendsen barostat [8] with 0.5-ps coupling constant and isothermal compressibility of 4.5e-5 bar^{-1}. Starting from the equilibrated structure, a production run of 500 ns was performed in the NVT ensemble for the unbiased MD simulation. For the restrained simulations, we obtained the backbone N-H RDCs for the inhibitor-free HIV-1PR from Roche *et al.* [9] and applied a linear potential (force constant 25,000 kJ/mol) between the experimental data and the RDCs calculated as averages over 4 independent simulations. Each replica was simulated for 125 ns in the NVT ensemble. We used the AMBER ff99SB*-ILDN force field [10, 11] for all simulations. First, the structure was minimized with the steepest descent algorithm to a tolerance of $10\,\mathrm{kJ\,mol^{-1}\,nm^{-1}}$ with restrained water molecules. Second, we simulated the system the for 5 ns in the NVT ensemble using the leap-frog integrator with a time step of 1 fs. A 1.2 nm cutoff was used for van der Waals interactions, with a force-switch modification at 1.0 nm. Coulomb interactions were treated with the particle-mesh Ewald method [12] with direct space cutoff of 1.2 nm. The temperature was set to 298 K using the v-rescale thermostat [13] with a 5 ps coupling constant. All the bonds involving hydrogen atoms were constrained using the LINCS algorithm [14].

T4 Lysozyme

The simulations of the L99A single mutant and the L99A-G113A-R119P triple mutant of T4 Lysozyme analysed in this study have been reported previously by Wang *et al.* [15]. The X-ray crystal structure of PDB code 3DMV was used as the initial configuration for the G states of both single and triple mutants. Simulations of the E states were started from chemical-shift-derived Rosetta-calculated structures, i.e. PDB code 2LCB and PDB code 2LC9 for single and triple mutant, respectively. Further simulations details can be found in the original article [15].

S1 Fig. Influence of Z cutoff on predicted DEER and PRE NMR data.

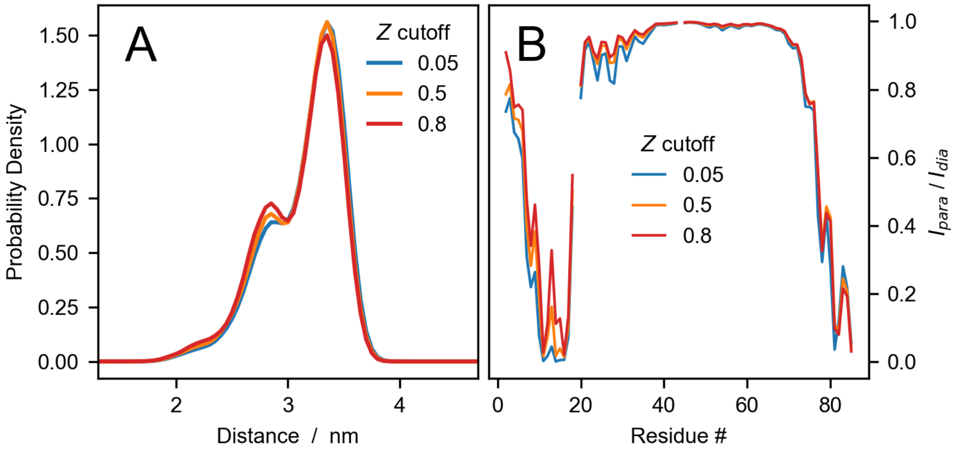

(A) DEER distance distributions calculated from RDC ensemble-biased MD simulations of HIV-1PR. (B) Predicted intensity ratios for ACBP spin-labeled at position 86 obtained from PDB code 1NTI with $\tau_c = 2\,\mathrm{ns}$, $\tau_t = 0.2\,\mathrm{ns}$, $t_d = 10\,\mathrm{ms}$, $R_2 = 12.6\,\mathrm{s}^{-1}$. DEER and PRE predictions are performed using three different cutoff values of the steric partition function, Z, namely 0.05 (blue lines), 0.5 (orange lines) and 0.8 (red lines).

S2 Fig. Comparison of DEER data from Torbeev *et al.* [6] with X-ray crystal structures deposited in the Protein Data Bank. This figure shows that although the DEER data is calculated from single X-ray crystal structures, the RLA results in multimodal distance distributions. For example, the K55-K55' separation between the ammonium groups in 1TW7 is ~ 3.6 nm, which is consistent with the semi-open conformation, however, the distances between the nitroxide groups of the MTSSL conformers span a wide range between 3.3 and 4.4 nm.

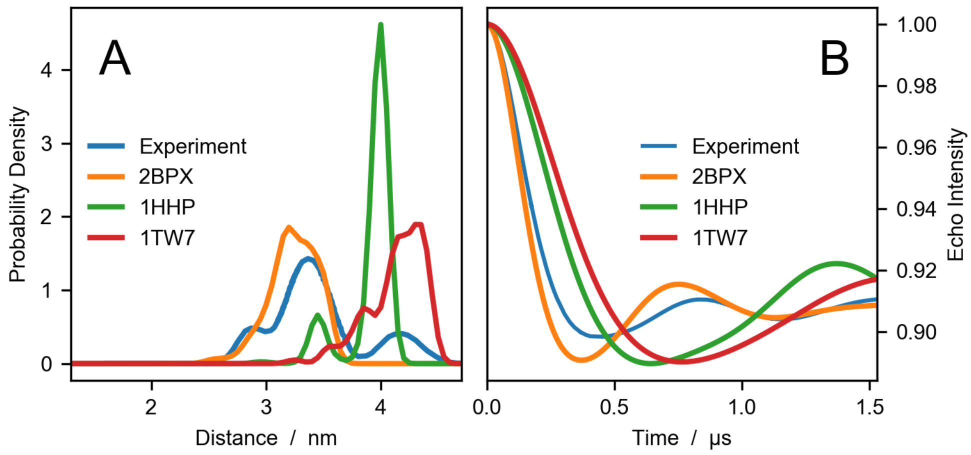

DEER distance distributions (A) and echo intensity curves (B) obtained by Torbeev *et al.* [6] from DEER experiments (blue), and calculated using X-ray crystal structures representative of closed (PDB code 2BPX, orange), semi-open (PDB code 1HHP, green) and wide-open (PDB code 1TW7, red) HIV-1PR conformations.

S3 Fig. Optimization of rotamer weights using a Bayesian/maximum entropy procedure. We use a Bayesian/maximum entropy (BME) procedure to find a modified set of rotamer weights for the MTSSL 175 K rotamer library [16], $\boldsymbol{w}$, which improves the agreement between predicted and experimental T109C–N140C $P(r)$'s for the single variant of T4L. The prediction is based on the 97:3 linear combination of $P(r)$'s from PDB codes 3DMV and 2LCB (corresponding to the populations of these two states) whereas the experimental data is from Lerch *et al.* [17]. Simulated annealing is used to minimize the cost function $\mathcal{L}(\boldsymbol{w}) = \chi^2(\boldsymbol{w}) - \theta S(\boldsymbol{w})$ where $\chi^2(\boldsymbol{w})$ is the sum of the squared differences between predicted and experimental $P(r)$'s, θ quantifies the confidence in the original weights, $\boldsymbol{w^O}$, and $S(\boldsymbol{w}) = -\sum_{i=1}^{N} w_i \ln \frac{w_i}{w_i^O}$ is the relative entropy defined as the negative of the Kullback-Leibler divergence between the modified weights of the $N = 46$ rotamers, w_i, and the initial w_i^O. The effective fraction of rotamers used in the reweighted ensemble, compared to the original library, is quantified as $\phi_{eff}(w) = \exp\left[S(w)\right]$. We scan various values of θ and select the weights obtained using $\theta = 4$ as the smallest modification resulting in a substantial decrease in χ^2. It is noteworthy that the change in weights has a lesser impact on the triple variant than on the single variant. Moreover, albeit being optimized against the T109C–N140C $P(r)$ of the single variant, the modified weights lead to an overall improvement in accuracy, with the average χ^2 over the four $P(r)$'s decreasing from 7.5 to 5.9.

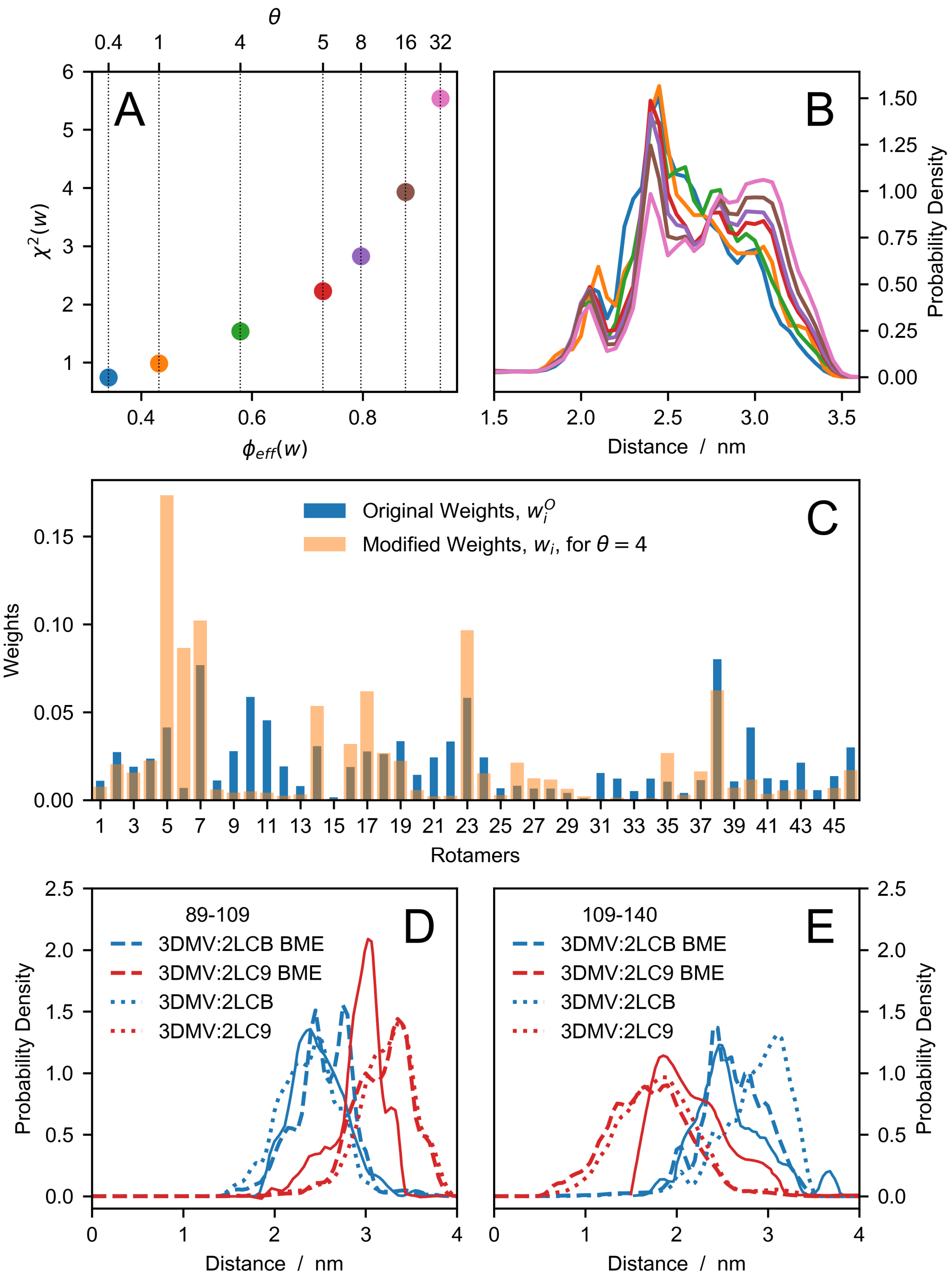

(A) χ^2 vs ϕ_{eff} for various values of the confidence parameter, θ. (B) Distance distributions calculated from PDB codes 3DMV and 2LCB, using optimized weights obtained for various θ values. (C) Original [16] and modified weights of the MTSSL 175 K rotamer library after BME reweighting with $\theta = 4$. DEER distance distributions for probe positions (D) D89C–T109C and (E) T109C–N140C of the single (blue) and the triple variant (red). Solid lines are the experimental data by Lerch *et al.* [17]; dotted and dashed lines are from PDB codes 3DMV, 2LC9 and 2LCB using the original and the BME-reweighted ($\theta = 4$) MTSSL 175 K rotamer library.

S4 Fig. Steric partition function quantifying the fitness of the rotamers at the spin-labeled site. While for most spin-label sites of ACBP the steric partition function, Z, varies between 1 and 1.5, for the placement of the probe at residue 86, Z drops below the cutoff of 0.05 proposed by Polyhach *et al.* [16] in five conformers out of 20. The significantly lower Z values indicate that residue 86 is in a tightly packed region in the protein structure and that spin-labeling position 86 can lead to structural deformations or changes in the populations of the conformational ensemble. This observation is consistent with stability experiments performed by Teilum *et al.* on wild type and spin-labeled mutants [18], showing that I86C is the least stable of the studied spin-labeled mutants. Although the RLA assumes that the overall protein conformation is unaffected by the presence of the spin-label, the case of the I86C mutant of ACBP highlights how DEER-PREdict can help to identify spin-labeled sites that are likely to violate this assumption in folded proteins.

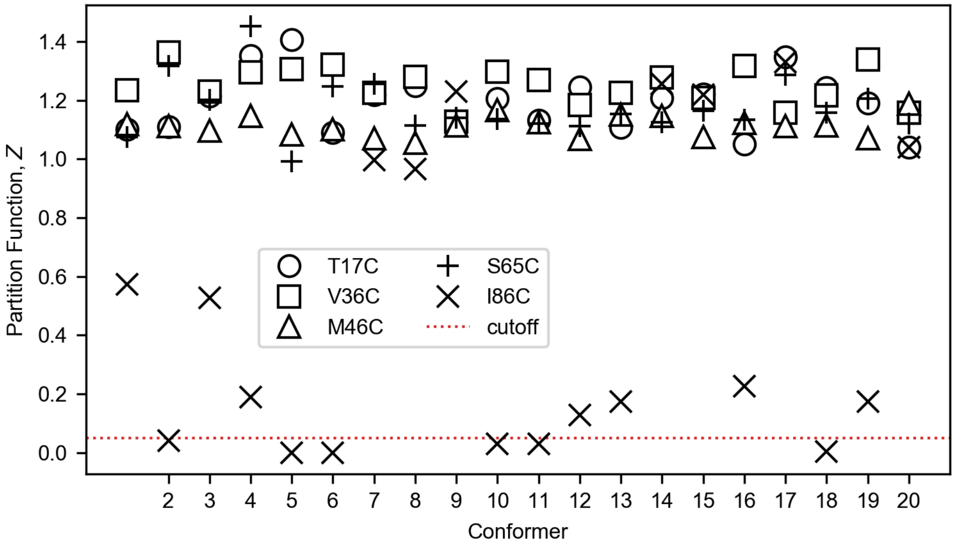

Steric partition function calculated from rotamer-protein van der Waals interactions for five spin-labeled mutants of ACBP. The horizontal dashed line indicates the cutoff used in the criterion for discarding protein conformations where the placement of the rotamer is characterized by steric clashes with the surrounding residues.

S5 Fig. Comparison with Cβ-based PRE Predictions The Cβ approximation may overestimate the effect of transient tertiary contacts on the PRE rates of ACBP, yielding experimentally consistent predictions only when the time scales for the reorientational dynamics is artificially made fast.

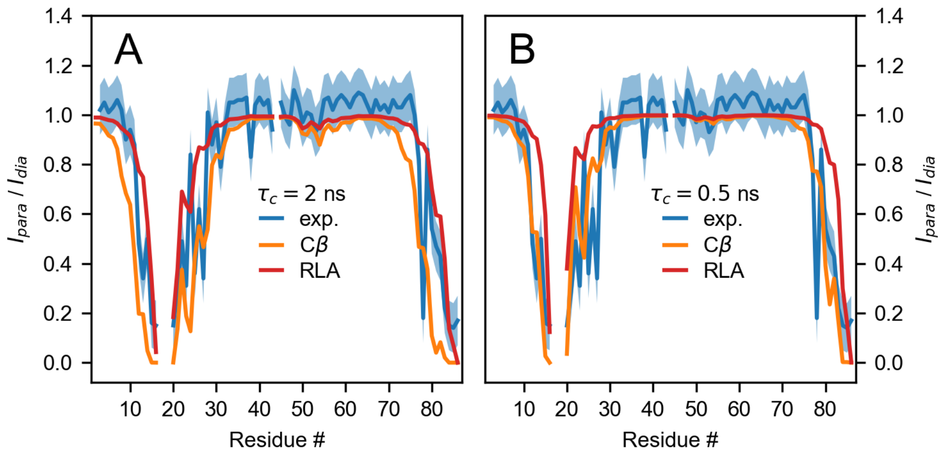

PRE intensity ratios for ACBP spin labeled at position 65 calculated for (A) $\tau_c = 2\,\mathrm{ns}$ and (B) $\tau_c = 0.5\,\mathrm{ns}$. Blue lines represent the experimental data [18], with the associated ±0.1 error shown by the blue shaded areas. Orange and red lines represent Cβ-based and RLA-based predictions, respectively.

S6 Fig. Dependence on τ_c of the RMSD between experimental and predicted PRE ratios of ACBP: Comparison of optimal τ_c values for RLA vs. Cβ-based approach. This figure illustrates the systematic time-scale difference when using explicit MTSSL probes instead of approximating the location of the unpaired electron with the position of the Cβ atom of the spin-labeled residue. Compared with the C_β-based approach, the RLA improves the accuracy in reproducing the experimental PRE data, as shown by the generally lower RMSD values. When using the RLA, the τ_c values that minimise the RMSD are found in the range of typical reorientational correlation time constants for proteins of ~ 100 residues (2–5 ns), and closer to the experimentally-derived value of 4 ns [18], whereas the Cβ-based approach underestimates the optimal τ_c.

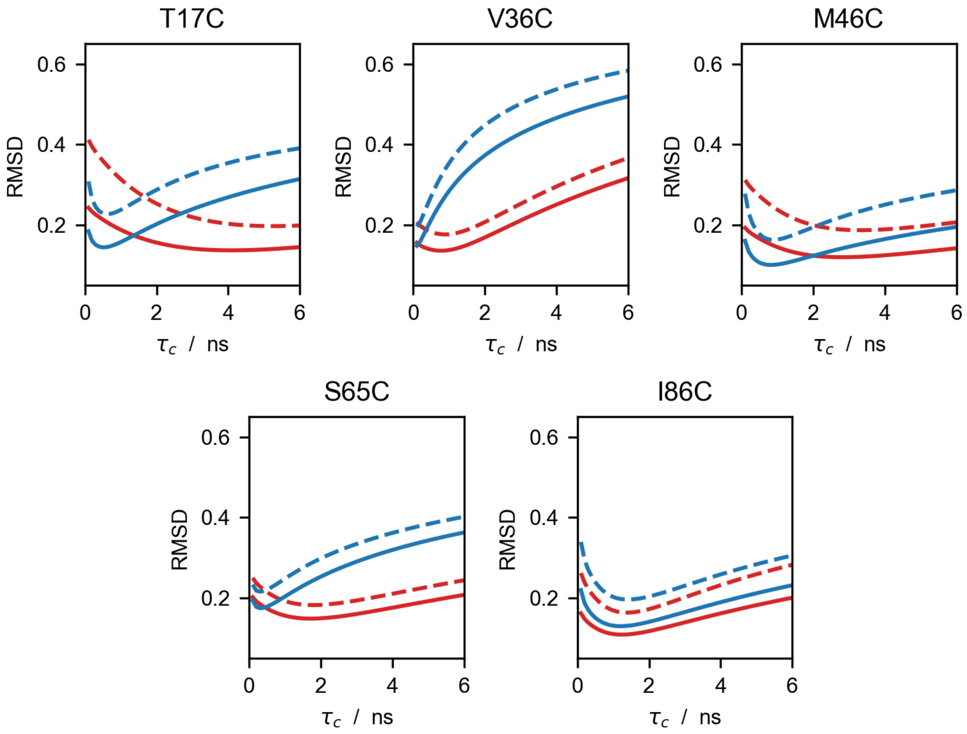

Red and blue lines are obtained using the RLA and approximating the electron location with the position of the Cβ atom, respectively. Solid and dashed lines represent the RMSD values calculated from all the data points and from intensity ratios in the dynamic range $0.1 < I_{para} \ / \ I_{dia} < 0.9$.

References

1. Abraham MJ, Murtola T, Schulz R, Páll S, Smith JC, Hess B, et al. GROMACS: High performance molecular simulations through multi-level parallelism from laptops to supercomputers. SoftwareX. 2015;1-2:19–25.

2. Tribello GA, Bonomi M, Branduardi D, Camilloni C, Bussi G. PLUMED 2: New feathers for an old bird. Computer Physics Communications. 2014;185(2):604–613.

3. Cavalli A, Camilloni C, Vendruscolo M. Molecular dynamics simulations with replica-averaged structural restraints generate structural ensembles according to the maximum entropy principle. J Chem Phys. 2013;138(9).

4. Camilloni C, Vendruscolo M. A tensor-free method for the structural and dynamical refinement of proteins using residual dipolar couplings. J Phys Chem B. 2015;119(3):653–661.

5. Sayer JM, Liu F, Ishima R, Weber IT, Louis JM. Effect of the active site D25N mutation on the structure, stability, and ligand binding of the mature HIV-1 protease. J Biol Chem. 2008;283(19):13459–13470.

6. Torbeev VY, Raghuraman H, Mandal K, Senapati S, Perozo E, Kent SBH. Dynamics of "Flap" Structures in Three HIV-1 Protease/Inhibitor Complexes Probed by Total Chemical Synthesis and Pulse-EPR Spectroscopy. J Am Chem Soc. 2009;131(3):884–885.

7. Blackburn ME, Veloro AM, Fanucci GE. Monitoring inhibitor-induced conformational population shifts in HIV-1 protease by pulsed EPR spectroscopy. Biochemistry. 2009;48(37):8765–8767.

8. Berendsen HJC, Postma JPM, van Gunsteren WF, DiNola A, Haak JR. Molecular dynamics with coupling to an external bath. The Journal of Chemical Physics. 1984;81(8):3684–3690.

9. Roche J, Louis JM, Bax A, Best RB. Pressure-induced structural transition of mature HIV-1 Protease from a combined NMR/MD simulation approach. Proteins. 2015;83(12):2117–2123.

10. Lindorff-Larsen K, Piana S, Palmo K, Maragakis P, Klepeis JL, Dror RO, et al. Improved side-chain torsion potentials for the Amber ff99SB protein force field. Proteins. 2010;78(8):1950–1958.

11. Best RB, Hummer G. Optimized Molecular Dynamics Force Fields Applied to the Helix−Coil Transition of Polypeptides. J Phys Chem B. 2009;113(26):9004–9015.

12. Darden T, York D, Pedersen L. Particle mesh Ewald: An N·log(N) method for Ewald sums in large systems. The Journal of Chemical Physics. 1993;98(12):10089–10092.

13. Bussi G, Donadio D, Parrinello M. Canonical Sampling Through Velocity Rescaling. J Chem Phys. 2007;126(1):014101.

14. Hess B. P-LINCS: A Parallel Linear Constraint Solver for Molecular Simulation. Journal of Chemical Theory and Computation. 2008;4(1):116–122.

15. Wang Y, Papaleo E, Lindorff-Larsen K. Mapping transiently formed and sparsely populated conformations on a complex energy landscape. eLife. 2016;5.

16. Polyhach Y, Bordignon E, Jeschke G. Rotamer libraries of spin labelled cysteines for protein studies. Phys Chem Chem Phys. 2011;13(6):2356–2366.

17. Lerch MT, López CJ, Yang Z, Kreitman MJ, Horwitz J, Hubbell WL. Structure-relaxation mechanism for the response of T4 lysozyme cavity mutants to hydrostatic pressure. Proc Natl Acad Sci U S A. 2015;112(19):E2437–46.

18. Teilum K, Kragelund BB, Poulsen FM. Transient Structure Formation in Unfolded Acyl-coenzyme A-binding Protein Observed by Site-directed Spin Labelling. J Mol Biol. 2002;324(2):349–357.
