# Supplementary figures and images for: DEER-PREdict: Software for efficient calculation of spin-labeling EPR and NMR data from conformational ensembles
Source: PLoS Comput Biol. 2021 Jan 22;17(1):e1008551. doi: 10.1371/journal.pcbi.1008551 (PMC7857587; doi:10.1371/journal.pcbi.1008551)

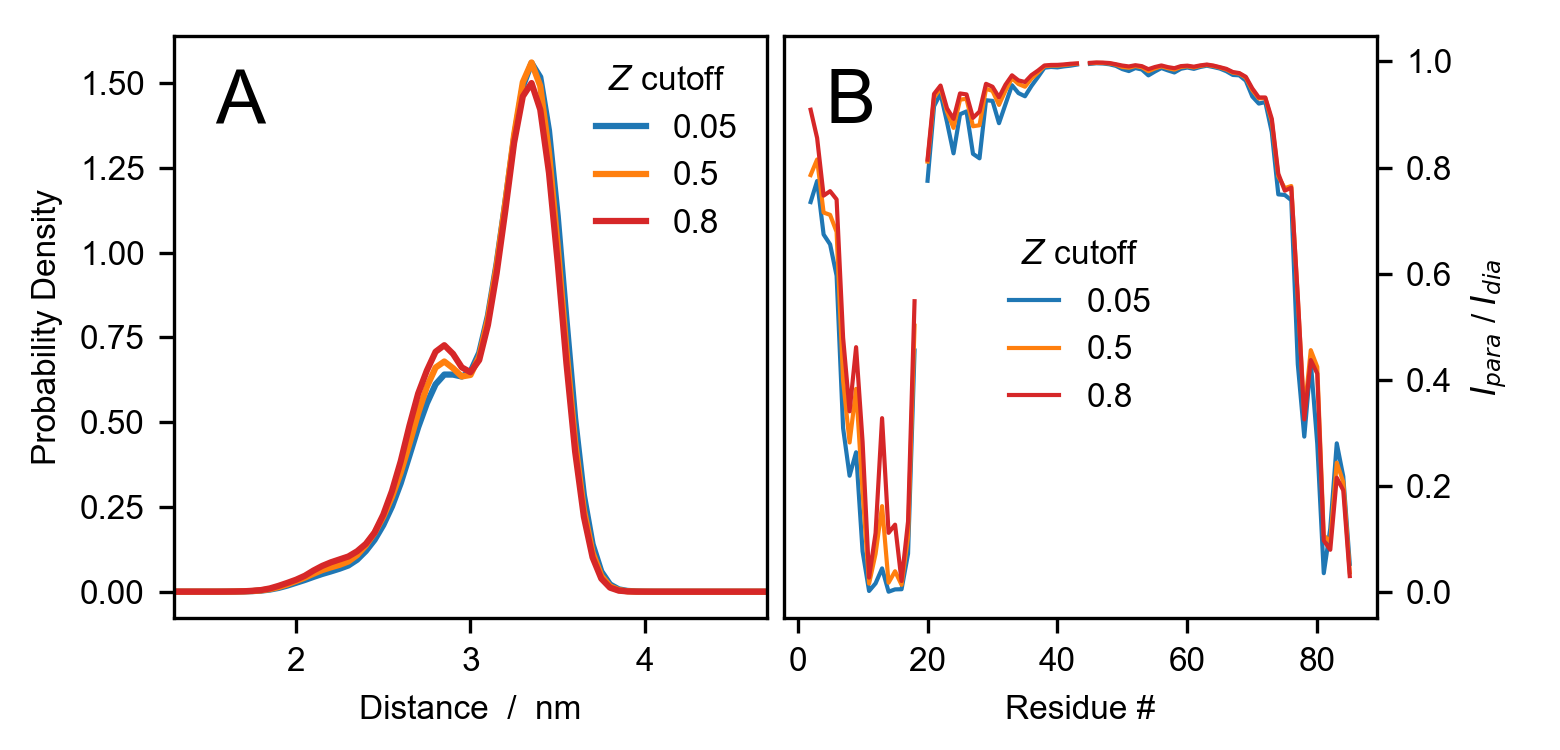

Supplement: S1 Fig — (A) DEER distance distributions calculated from RDC ensemble-biased MD simulations of HIV-1PR. (B) Predicted intensity ratios for ACBP spin-labeled at position 86 obtained from PDB code 1NTI with τc = 2 ns, τt = 0.2 ns, td = 10 ms, R2 = 12.6 s−1. DEER and PRE predictions are performed using three different cutoff values of the steric partition function, Z, namely 0.05 (blue lines), 0.5 (orange lines) and 0.8 (red lines). (TIF) [file pcbi.1008551.s002.tif]

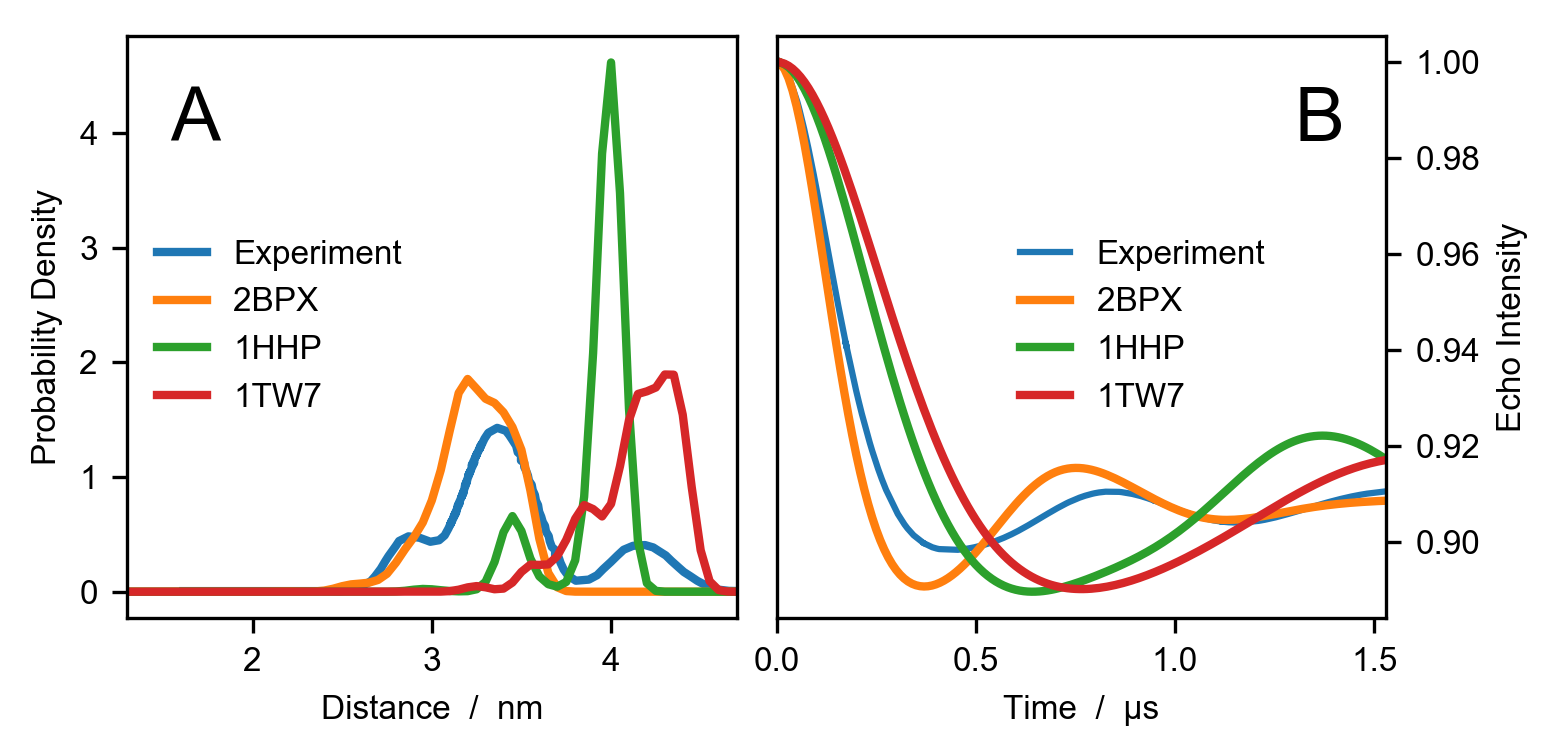

Supplement: S2 Fig — DEER distance distributions (A) and echo intensity curves (B) obtained by Torbeev et al. [44] from DEER experiments (blue), and calculated using X-ray crystal structures representative of closed (PDB code 2BPX, orange), semi-open (PDB code 1HHP, green) and wide-open (PDB code 1TW7, red) HIV-1PR conformations. (TIF) [file pcbi.1008551.s003.tif]

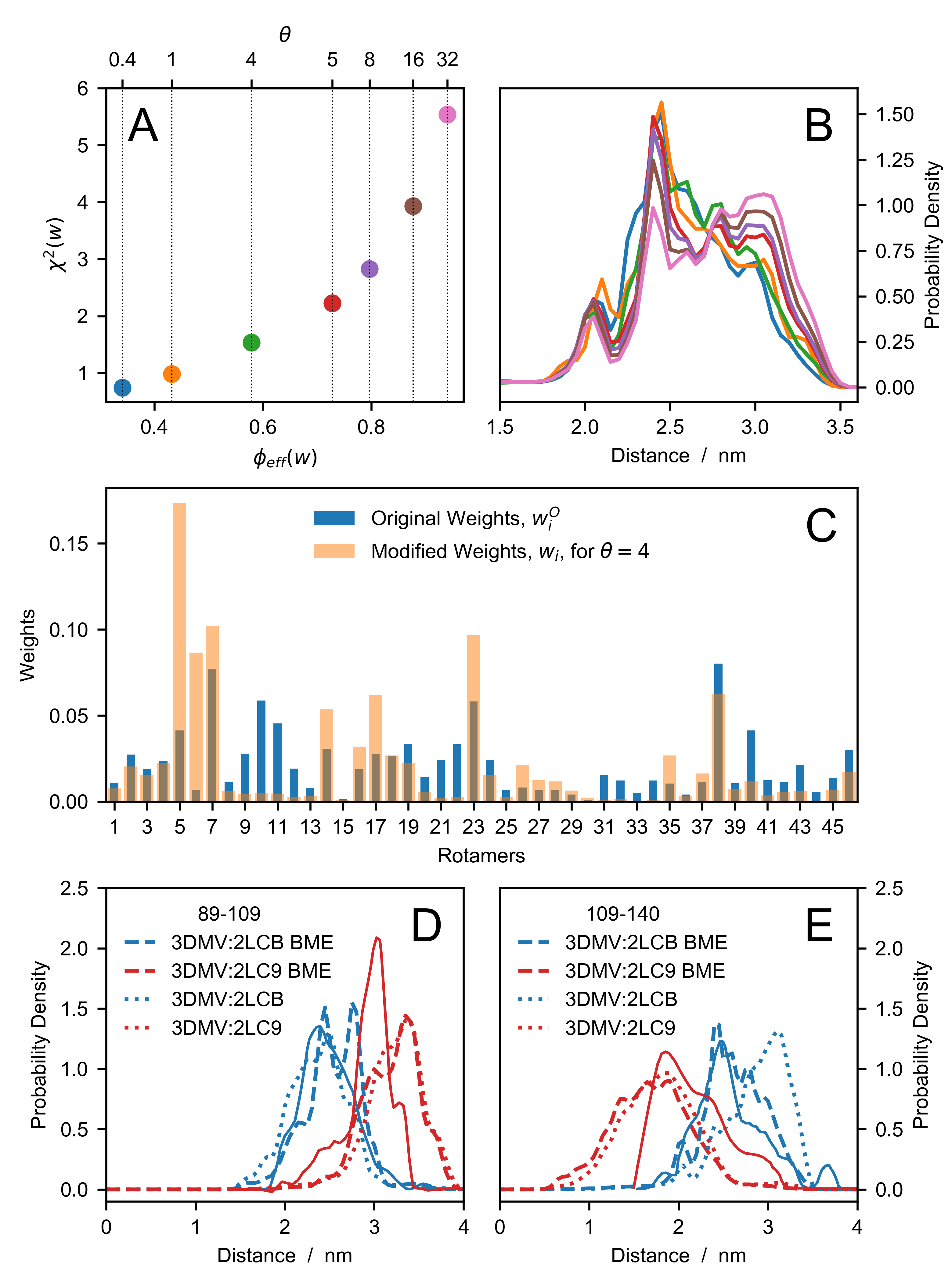

Supplement: S3 Fig — (A) χ2 vs φeff for various values of the confidence parameter, θ. (B) Distance distributions calculated from PDB codes 3DMV and 2LCB, using optimized weights obtained for various θ values. (C) Original [24] and modified weights of the MTSSL 175 K rotamer library after BME reweighting with θ = 4. DEER distance distributions for probe positions (D) D89C–T109C and (E) T109C–N140C of the single (blue) and the triple variant (red). Solid lines are the experimental data by Lerch et al. [83]; dotted and dashed lines are from PDB codes 3DMV, 2LC9 and 2LCB using the original and the BME-reweighted (θ = 4) MTSSL 175 K rotamer library. (TIF) [file pcbi.1008551.s004.tif]

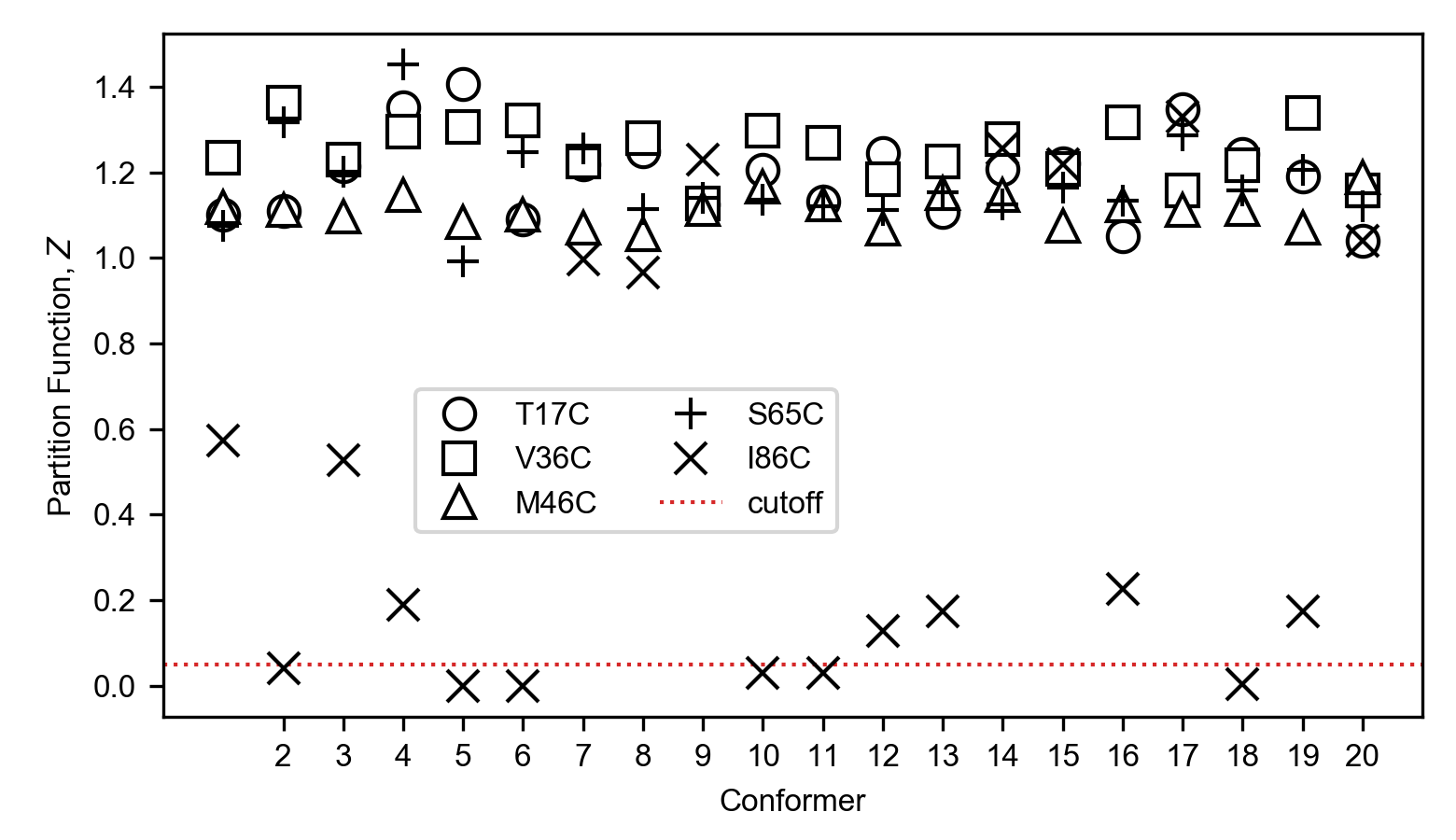

Supplement: S4 Fig — Steric partition function calculated from rotamer-protein van der Waals interactions for five spin-labeled mutants of ACBP. The horizontal dashed line indicates the cutoff used in the criterion for discarding protein conformations where the placement of the rotamer is characterized by steric clashes with the surrounding residues. (TIF) [file pcbi.1008551.s005.tif]

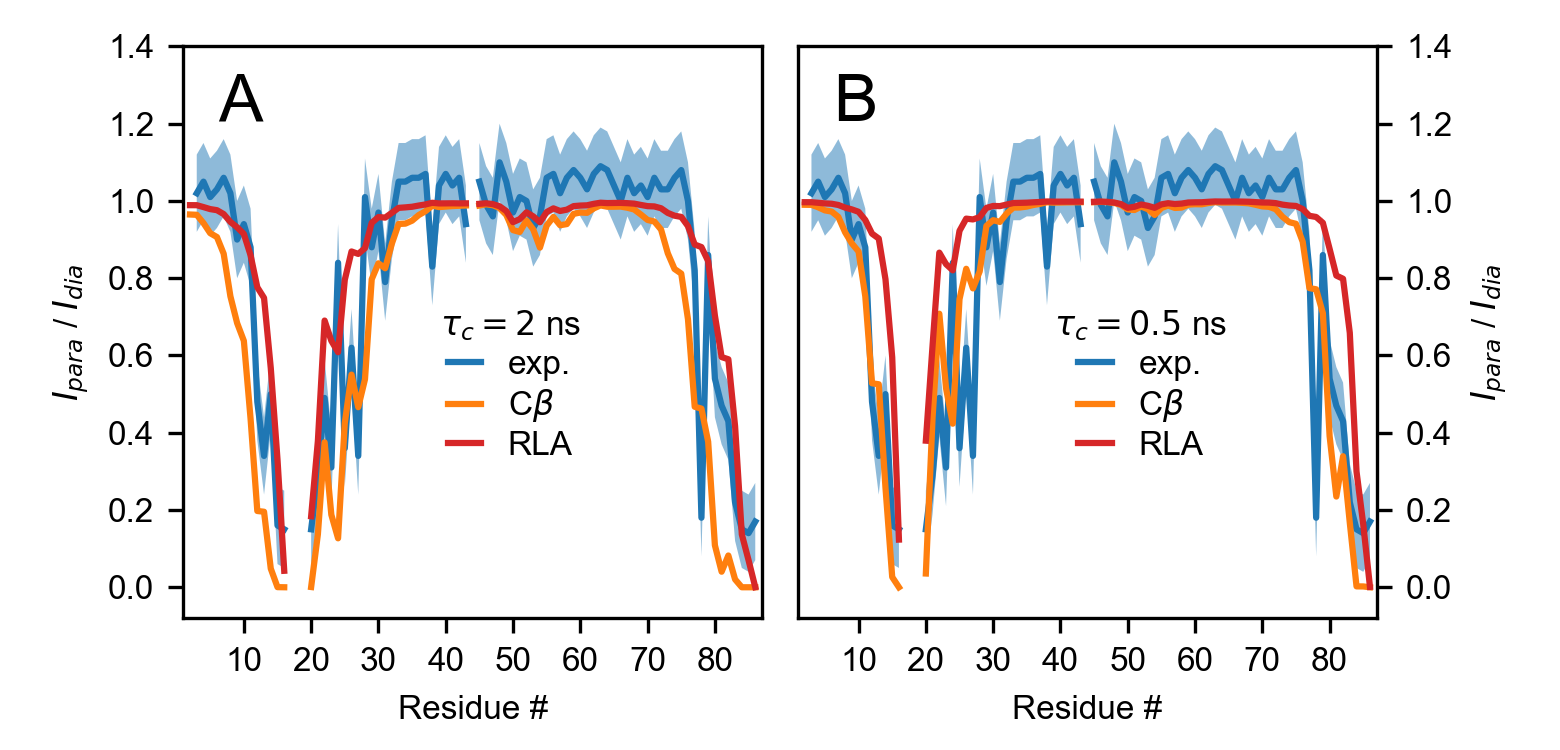

Supplement: S5 Fig — PRE intensity ratios for ACBP spin labeled at position 65 calculated for (A) τc = 2 ns and (B) τc = 0.5 ns. Blue lines represent the experimental data [53], with the associated ±0.1 error shown by the blue shaded areas. Orange and red lines represent Cβ-based and RLA-based predictions, respectively. (TIF) [file pcbi.1008551.s006.tif]

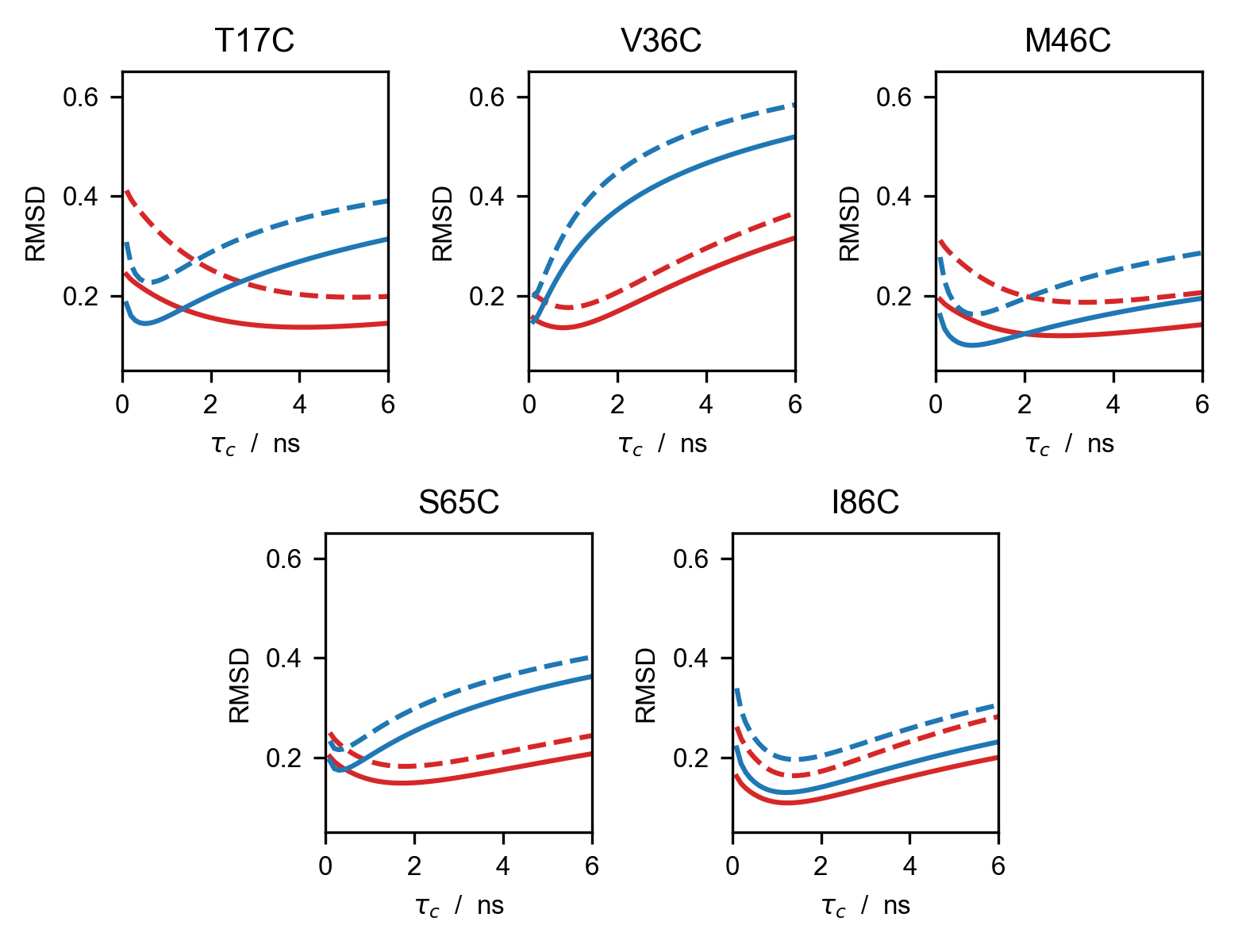

Supplement: S6 Fig — Red and blue lines are obtained using the RLA and approximating the electron location with the position of the Cβ atom, respectively. Solid and dashed lines represent the RMSD values calculated from all the data points and from intensity ratios in the dynamic range 0.1 < Ipara / Idia < 0.9. (TIF) [file pcbi.1008551.s007.tif]
