# Supplementary material for: DEER-PREdict: Software for efficient calculation of spin-labeling EPR and NMR data from conformational ensembles
Source: PLoS Comput Biol. 2021 Jan 22;17(1):e1008551. doi: 10.1371/journal.pcbi.1008551 (PMC7857587; doi:10.1371/journal.pcbi.1008551)
Supplement: Attachment — Submitted filename: __DEERpredict__response-letter__2__lindorff__2020__.docx [file pcbi.1008551.s008.docx]

| Prof. Dina Schneidman, Software Editor, PLOS Computational Biology |  |
| --- | --- |
| Revision of manuscript (PCOMPBIOL-D-20-01460) | 7th nov 2020 |

Thank you very much for sending us the reviewer comments on our paper “***DEER-PREdict: Software for Efficient Calculation of Spin-Labeling EPR and NMR Data from Conformational Ensemble”***.

We were pleased with the positive response, and with the detailed and very useful comments and questions. We have now addressed these in a revised version of the manuscript. A point-by-point response to the comments/questions follows this letter.

Yours sincerely,

Prof. Kresten Lindorff-Larsen

**Reviewer 1**

This manuscript reports on a very useful software for predicting distance distributions for spin label pairs as well as experimental DEER/PELDOR data and paramagnetic relaxation enhancement (PRE) data from MD trajectories. The software is implemented in a user-friendly way and is computationally efficient. Especially for PRE it enables better predictions than are currently made by most practitioners in the field, as demonstrated in the Supporting Information. In general, the performance is nicely illustrated on the three application examples, although there is some unexplained discrepancy for one site pair and one mutant for T4 Lysozyme (see below). The manuscript is well written and concise. I recommend minor revision, taking into account the suggestions below.

We thank Prof. Jeschke for his very insightful and detailed comments on our paper, and for highlighting the utility of our work.

1. In line 14, you quote a distance range from ~2 to ~6 nm. This is outdated information. A 2012 review (doi: 10.1146/annurev-physchem-032511-143716) already quoted 8 nm without protein deuteration and 11.5 nm with protein deuteration for soluble proteins. A 2016 paper (doi: 10.1002/anie.201609617) demonstrated 16 nm on a fully deuterated protein, admittedly in an exceptional case. In any case, the 6 nm limit has been surpassed in many application examples.

Thanks for pointing this out. In the revised manuscript we have modified the range of distances that can be probed in DEER experiments in the text (lines 14–15) and cited doi: 10.1146/annurev-physchem-032511-143716 (Ref. 10) and doi: 10.1002/anie.201609617 (Ref. 9)

2. Line 73: It would be useful to specify what information must be included in a rotamer library

We now specify that a rotamer library comprises a PDB topology file, a DCD trajectory file and a text file containing the rotamer weights (lines 75–78).

3. Line 105: I believe that Ref. 34 does not relate to MMM.

We apologize for the error and have moved ref. 34 (ref. 36 in the revised version) to line 111 where DeerLab is mentioned explicitly as an example of a package to extract electron-electron distance distributions from DEER experiments

4. Line 118: Please specify the low pass filter. It leads to some broadening of the distance distribution, which is fine, as the rotamer approach neglects some contributions to conformational distribution, but this broadening should not be excessive.

We now specify the low pass filter (Eq. 2 at line 125), report the standard deviation of the filter (line 131), and made it possible for the user to specify this parameter through the option *filter_stdev* of the *run* function in the *DEERpredict* class.

5. Below Eq (3): Please specify the kernel (minimum and maximum r, r increment, maximum t, t increment) or state that these values can be set by the user and provide recommended settings (especially for the increments).

We now have specified the kernel (lines 140–142) by mentioning the default parameters for *r* and *t*. We also modified the code to make it possible for the user to override the default values for *r_max_*, *t_min_*, *t_max_* and *dt*.

6. The result of Eq. (5) cannot be directly compared to the experimental DEER trace. It describes the so-called form factor after correction for the intermolecular background. Either state this (and give a reference) or provide an explanation (and equations) for including this background. In general, it would be useful to refer to pre-processing that is necessary. Descriptions can be found in either Ref. 34 (a Python package amenable to pipelining with your software) or the older doi: 10.1007/BF03166213.

Thank you for raising the clarity issue in our description of how to compare Eq. 6 (previously Eq. 5) with the experimental DEER trace. In the revised version, we clarify that Eq. 6 can be compared with the time trace after applying the correction for the intermolecular background and cite ref. 36 (previously ref. 34) as well as doi: 10.1007/BF03166213 (ref. 42)

7. Line 144: “paramagnetic spin number” could be read as number of electron spins, but it is the electron spin quantum number.

We have replaced “paramagnetic spin number” by “electron spin quantum number” at line 162

8. Line 160: In reality, the jump rates between rotamers vary strongly (see Ref. 15). You might want to point out that this PRE model with a single correlation time is an approximation. It would also be useful to mention the time scale for such “jumps” implied by the results in Ref. 15.

Thank you for highlighting that we were not clear on this important point, with which we agree. In the revised manuscript we now describe the jump model assumes equal transition probabilities between dihedral conformations and mention the range of residence times between jumps (lines 178–181), as implied in the results of ref. 17 (previously ref. 15).

9. Results for T4L: For site pair 89-109 (Fig. 3A) the agreement of metadynamics simulations with experiments is as good as one can expect. For site pair 109-140, the experimental result for the triple mutant (red in Fig. 3B) probably misses the shorter distances predicted by metadynamics because they fall outside the DEER range (you may want to comment on that). However, for mutant L99A the metadynamics simulation is clearly off, well beyond expected combined error of experiment and rotamer approach, whereas the prediction from PDB 2LCB fits experimental data almost perfectly. Either this is a freezing-related problem or a problem with the metadynamics simulation for this mutant. In any case, the discrepancy needs to be mentioned and discussed to some extent.

We thank the reviewer for pointing out these discrepancies between predicted and experimental *P(r)* for the spin-label pair 109-140 in both the L99A and triple mutant of T4 lysozyme.

Regarding the triple mutant, we now mention in the revised manuscript that the short distances lie below the range probed by DEER experiments (lines 325--328).

To address the discrepancy between predicted and experimental distance distribution for the 109-140 pair in the triple mutant we follow the approach described in doi: 10.1021/acs.jpclett.8b02439 and modify the weights of the MTSSL 175 K rotamer library through a Bayesian/maximum entropy procedure (lines 328–332 and fig. S3). The result of the optimization highlights that a small change in the rotamer weights can substantially improve the agreement with the experimental data, suggesting that the discrepancy could be attributed to the rotamer library rather than to the protein conformational ensemble.

Typos:
Line 5: “spacial resolution” should read “spatial resolution”

Thank you for spotting this typo. We have corrected it in the revised manuscript.

**Reviewer 2**

The authors introduce DEER-PREdict, a software to calculate EPR and NMR data from computational ensembles of spin-label biomolecules. DEER-PREdict is an important methodological advance for employing data from EPR (DEER) and NMR (PRE) experiments in integrative modelling of biomolecules.

It is very useful that the DEER-PREdict provides implementations of the calculation of the DEER and PRE signals. Direct comparison to the experimental data are vital when assessing ensembles from computational modelling or trying to understand patterns in the experimental data.

The examples in the manuscript are highly relevant and help to illustrate the power of this approach. E.g., the agreement shown for the PRE data of ACBP (in Fig. 4) shows that very good agreement can obtained and that taking the spin labels into account captures features that otherwise cannot be reproduced (Fig. S4).

DEER-PREdict is easy to install and use. The software is nicely written and the code is straightforward to understand. Comments in the code further aid understanding. Importantly, automatic tests ensure that further modifications do not break the code.

DERR-PREdict will become a very useful tool for structural biologists and biophysicists.

We the reviewer for highlighting the utility of our work.
